# Genetic and clinical observational evidence for associations between sleep disorders and psychiatric disorders

**DOI:** 10.3389/fpsyt.2026.1845263

**Published:** 2026-08-14

**Authors:** Xin Wu, Mei Chang, Bingyi Song, Jiaxuan Li, Longyuan Li, Ziqian Yin, Zhouqing Chen, Zhong Wang

**Affiliations:** First Affiliated Hospital of Soochow University, Suzhou, China

**Keywords:** attention deficit hyperactivity disorder, insomnia, major depressive disorder, Mendelian randomization study, psychiatric disorders, retrospective study

## Abstract

**Background:**

The relationship between sleep disorders and psychiatric disorders has been extensively studied in epidemiological research. However, whether specific sleep-related traits are associated with psychiatric disorders through potential causal mechanisms remains incompletely understood.

**Methods:**

This study employed a two-sample Mendelian randomization (MR) analysis method to investigate potential causal associations between six sleep-related traits and eleven psychiatric disorders. MR analyses were performed using inverse variance weighting (IVW), weighted median, and MR-Egger methods. Cochran’s Q test was used to assess heterogeneity, while MR-Egger intercept analysis and MR-PRESSO (Mendelian Randomization Pleiotropy RESidual Sum and Outlie) were applied to evaluate potential horizontal pleiotropy. In addition, a retrospective observational study was conducted using clinical data from the First Affiliated Hospital of Soochow University between November 2023 and December 2025. Logistic regression analysis was performed to assess the association between sleep disorders and psychiatric disorders.

**Results:**

MR analyses identified potential associations between sleep-related traits and psychiatric disorders. Genetically predicted insomnia was associated with an increased risk of major depressive disorder (OR = 2.22, 95% CI: 1.66–2.97, P < 0.001). In the retrospective clinical cohort, individuals with sleep disorders exhibited a higher prevalence of psychiatric disorders (adjusted OR = 1.71, 95% CI: 1.09–2.69, P = 0.020). The consistency between genetic evidence and clinical observational findings provides complementary support for the clinical relevance of the observed association.

**Conclusion:**

By integrating MR analyses with retrospective clinical observational data, this study provides genetic and clinical evidence supporting an association between sleep disorders and psychiatric disorders. These findings suggest that sleep disturbances may represent potential modifiable factors associated with mental health outcomes and highlight the importance of sleep-related interventions in psychiatric disease prevention and management.

## 1 Introduction

Psychiatric disorders affect approximately 30% of individuals worldwide and represent one of the leading contributors to global disease burden, accounting for 13% of years lived with disability and 32% of years lived with disability overall (1). As a highly heterogeneous group of disorders, the onset and progression of psychiatric disorders are influenced by complex interactions between biological, genetic, and environmental factors. A growing body of epidemiological evidence indicates that a range of psychiatric conditions, including schizophrenia (SCZ), bipolar disorder (BD), major depressive disorder (MDD), anxiety disorders (ANX), and stress-related disorders, are associated with sleep patterns (2). Interventions targeting sleep disturbances and circadian rhythm dysfunction, including pharmacological, psychosocial, and chronobiological approaches, have shown potential benefits in the prevention and management of psychiatric disorders (3, 4).

Epidemiological studies further suggest that sleep disorders are associated not only with the occurrence of various psychiatric disorders but also with their clinical progression and prognosis (5, 6). Increasing evidence suggests that circadian rhythm disturbances, as a subtype of sleep-related abnormalities, may contribute to the development of psychiatric disorders or at least hold significant pathophysiological significance. For example, sleep disturbances and abnormal circadian rhythm patterns are commonly observed among individuals with BD and anorexia nervosa (AN) (3, 6). Previous studies have reported an association between insomnia-related genetic susceptibility and increased risk of AN (6). In addition, interventions targeting insomnia, such as cognitive behavioral therapy for insomnia, have been shown to improve depressive symptoms (7). These findings provide evidence supporting the potential involvement of sleep disorders in the development and progression of psychiatric disorders.

At the clinical level, sleep disorders have been reported to be associated with increased suicide risk in later life and with the occurrence of major depressive disorder8(p3) and SCZ (4). A retrospective study conducted through the Kingston Health Sciences Centre (KHSC) Sleep Laboratory included 74 participants, comprising 37 with sleep disorders and 37 with early-onset psychosis, for statistical analysis. Within the sleep disorder cohort, a higher prevalence of psychiatric diagnoses and early-onset psychotic symptoms was observed (9). Disturbed sleep may negatively influence mood and emotional wellbeing, precede the emergence of psychiatric symptoms, and potentially affect treatment outcomes (4).However, despite substantial observational studies and clinical evidence suggesting an association between sleep disorders and psychiatric disorders, these study designs cannot fully exclude residual confounding and reverse causality. MR is an analytical approach that utilizes single nucleotide polymorphisms (SNPs) as instrumental variables to investigate potential causal effects of exposures on outcomes. Because genetic variants are randomly allocated during meiosis, MR analysis may reduce the influence of confounding factors and reverse causality, thereby providing evidence regarding potential causal relationships under specific assumptions (10, 11). This study aims to investigate potential causal associations between sleep disorders and psychiatric disorders using MR analysis and to further evaluate their clinical relevance through a retrospective observational study.

## 2 Method

### 2.1 Study design

MR analyses were performed to investigate potential causal associations between six sleep-related traits, including sleep duration, insomnia, daytime dozing, napping, chronotype, and snoring, and eleven psychiatric disorders, including autism spectrum disorder (ASD), SCZ, obsessive-compulsive disorder (OCD), MDD, BD, AN, ANX, panic disorder, post-traumatic stress disorder (PTSD), ADHD, and delusional disorder. GWAS (Genome-Wide Association Study) summary statistics for exposure and outcome traits were obtained from publicly available databases, including the Open GWAS database and FinnGen. All procedures involving human participants were conducted in accordance with the Declaration of Helsinki and relevant ethical guidelines. The original GWAS studies included appropriate ethical approvals from their respective institutional review boards.

**Figure 1:**
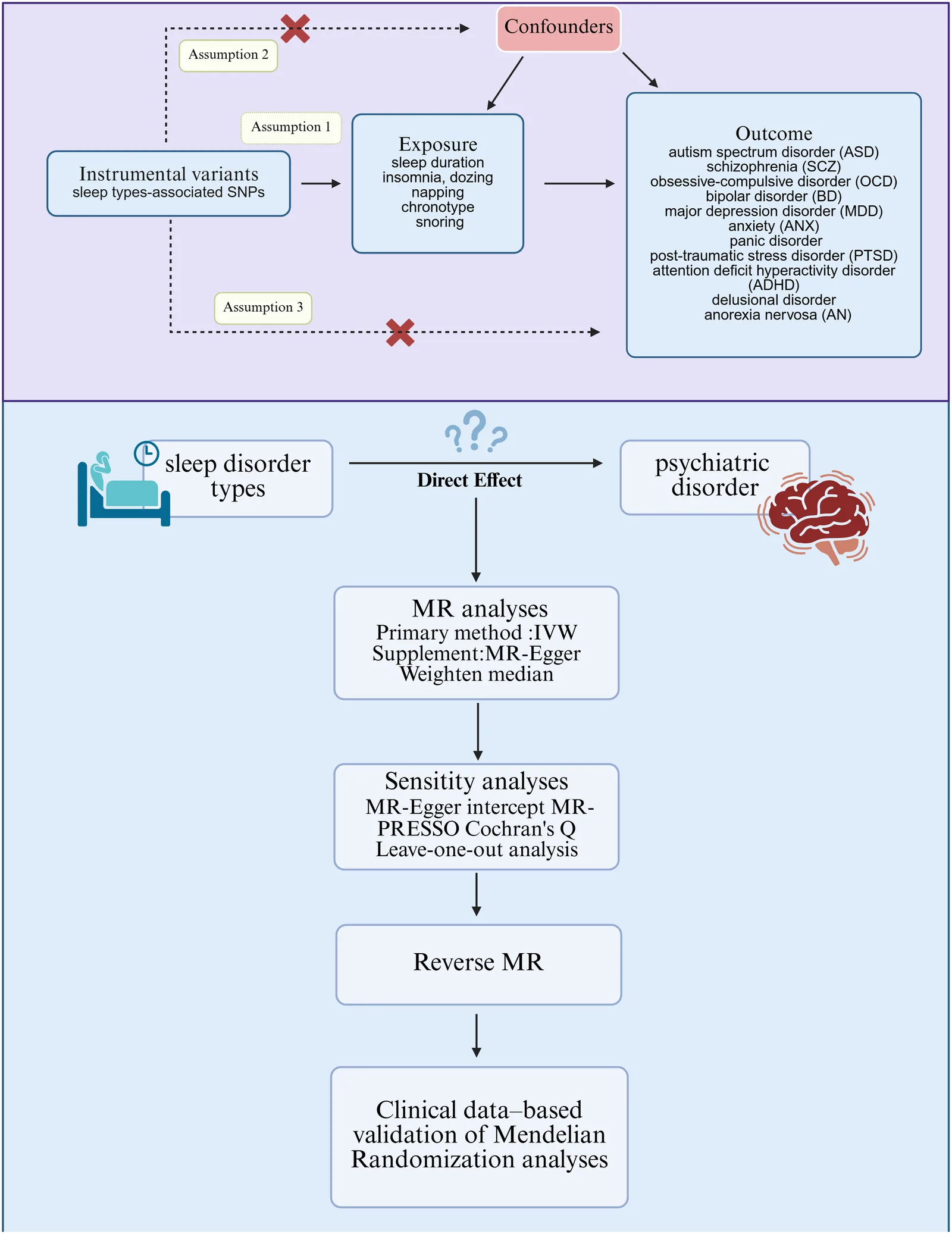
Overview of the MR study design and clinical validation. Genetic variants associated with sleep-related traits were used as instrumental variables to assess causal relationships with psychiatric disorders using Mendelian randomization, complemented by bidirectional analyses, sensitivity tests, and validation using clinical observational data.

The MR design is based on the following assumptions: (1) genetic variants are strongly associated with sleep-related traits and satisfy genome-wide significance criteria; (2) the genetic variants used are not associated with any confounding factors; (3) genetic variants influence psychiatric outcomes only through the corresponding sleep-related traits and not through alternative biological pathways (12, 13). Finally, we conducted a retrospective observational study in a single-center clinical cohort to evaluate the clinical association between sleep disorders and psychiatric conditions and provide complementary clinical evidence (**Figure 1**).

### 2.2 Data sources

The datasets used in this study were retrieved from publicly available GWAS datasets. Specifically, publicly available GWAS summary datasets, primarily derived from European populations, were used, including six sleep-related traits and eleven psychiatric disorders. These necessary data are sourced from the OPEN GWAS website (https://gwas.mrcieu.ac.uk) and FinnGen database (14). Detailed data sources, including GWAS IDs, population, and sample sizes, are provided in **Supplementary Tables 1**, **2**.

### 2.3 The selection of IVs

In MR analyses, IVs are genetic variants used to estimate the potential causal effect of an exposure on an outcome. IVs are usually genetic variations, the most commonly used of which are SNPs. Linkage disequilibrium (LD) clumping was performed using a threshold of R² < 0.001 and a clumping window of 10,000 kb to ensure independence among selected SNPs. Genetic variants showing genome-wide significant associations with the outcome (p < 5 × 10^-8^) were excluded to minimize potential bias due to reverse causation or horizontal pleiotropy. The F statistic was calculated to evaluate instrument strength. An F statistic greater than 10 is commonly considered indicative of sufficient instrument strength and helps reduce the potential bias caused by weak instrumental variables (15).

### 2.4 MR analysis

In this MR study, the inverse-variance weighted (IVW) method was used as the primary analytical approach to estimate the association between genetically predicted sleep-related traits and psychiatric outcomes. The IVW method provides an efficient overall estimate when all instrumental variables are valid or when horizontal pleiotropic effects are balanced (16). Heterogeneity among SNP-specific estimates was assessed using Cochran’s Q statistic in both the IVW and MR-Egger models, with a P value <0.05 indicating evidence of heterogeneity. MR-Egger regression was used as a sensitivity analysis to evaluate the potential influence of directional horizontal pleiotropy. The MR-Egger intercept was used to test for directional pleiotropy, whereas the slope provided an alternative estimate of the exposure–outcome association. Although MR-Egger regression can provide estimates that are less sensitive to certain forms of directional pleiotropy, its statistical power is generally lower than that of the IVW method, and its validity depends on the Instrument Strength Independent of Direct Effect assumption (17). The weighted median method provides a consistent estimate when at least 50% of the total weight is contributed by valid instrumental variables and may offer greater statistical power than MR-Egger regression under certain conditions (16, 18).MR-PRESSO analysis was additionally performed to identify potential outlier SNPs and evaluate horizontal pleiotropy using the global test. When outliers were detected, outlier-corrected estimates were examined (19). Because these MR methods rely on different assumptions, consistency in the direction and magnitude of estimates across methods was considered supportive of the robustness of the observed associations, although such consistency does not by itself establish causality (8). Bidirectional MR analyses were performed, with sleep-related traits treated as exposures and psychiatric disorders as outcomes in the forward analyses, and psychiatric disorders treated as exposures and sleep-related traits as outcomes in the reverse analyses. To account for multiple testing, raw P values from the primary IVW analyses were adjusted using the Benjamini–Hochberg false discovery rate (FDR) procedure across all tested exposure–outcome combinations. Associations with an FDR-adjusted P value (q value) <0.05 were considered statistically significant, whereas associations with a raw P value <0.05 but an FDR-adjusted P value ≥0.05 were considered nominally significant. Leave-one-out analyses were performed by sequentially removing each SNP to determine whether the overall IVW estimate was disproportionately influenced by any single instrumental variable. Scatter plots were generated to display the associations of individual SNPs with the exposure and outcome, whereas forest plots were used to present SNP-specific estimates and their contributions to the overall MR estimate. Reverse MR analyses followed the same procedures for instrument selection, harmonization, primary estimation, multiple-testing correction, and sensitivity analyses as the forward MR analyses.

All statistical analyses were conducted using R software (version 3.4.1). Data harmonization and MR analyses investigating potential causal associations between sleep-related traits and psychiatric disorders were performed using the TwoSampleMR package. The design, analysis, and reporting of the MR component followed the recommendations of the STROBE-MR (Strengthening the Reporting of Observational Studies in Epidemiology Using Mendelian Randomization) guideline. MR-PRESSO analyses, including the global test for horizontal pleiotropy and the identification of potential outlier SNPs, were conducted using the MR-PRESSO package.

### 2.5 Clinical observational analysis

To complement the genetic evidence obtained from the MR analyses, we conducted a retrospective observational analysis using clinical data to evaluate the association between sleep disorders and psychiatric disorders in a real-world clinical setting. The retrospective observational component received ethical approval from the Ethics Committee of the First Affiliated Hospital of Soochow University (approval number: 2026007). Depressive symptoms, anxiety symptoms, and insomnia severity were assessed using the Patient Health Questionnaire-9 (PHQ-9), Generalized Anxiety Disorder-7 (GAD-7), and Insomnia Severity Index (ISI), respectively. This retrospective observational study included 350 hospitalized participants from the First Affiliated Hospital of Soochow University between November 2023 and December 2025. The presence of a psychiatric disorder was coded as a binary outcome variable (present/absent) according to the predefined clinical assessment criteria. Sex and age were included as covariates in the multivariable model. Age was categorized into three groups: <25 years, 25–60 years, and >60 years. Baseline characteristics were summarized according to psychiatric disorder status and reported as frequencies and column percentages for categorical variables, in accordance with the STROBE (Strengthening the Reporting of Observational Studies in Epidemiology) reporting guideline (20). Differences in categorical variables between participants with and without psychiatric disorders were assessed using Pearson’s chi-square test. Because age was analyzed as an ordered categorical variable, a chi-square test for trend was additionally performed across the three age groups. The unadjusted association between sleep disorder status and psychiatric disorder status was initially examined using a 2 × 2 contingency table, from which the crude odds ratio (OR) and corresponding 95% confidence interval (CI) were calculated. Multivariable binary logistic regression was subsequently performed to estimate the adjusted association between sleep disorder status and psychiatric disorder status, with sleep disorder status entered as the primary independent variable and sex and age category included as covariates. Adjusted ORs and corresponding 95% CIs were reported (21, 22). All statistical tests were two-sided, and a P value <0.05 was considered statistically significant for the clinical observational analysis.

## 3 Results

### 3.1 The results of MR analysis

This study employed forward MR analyses, reverse MR analyses, and FDR correction to evaluate associations between sleep-related traits and psychiatric disorders while accounting for multiple testing.

**Figure 2:**
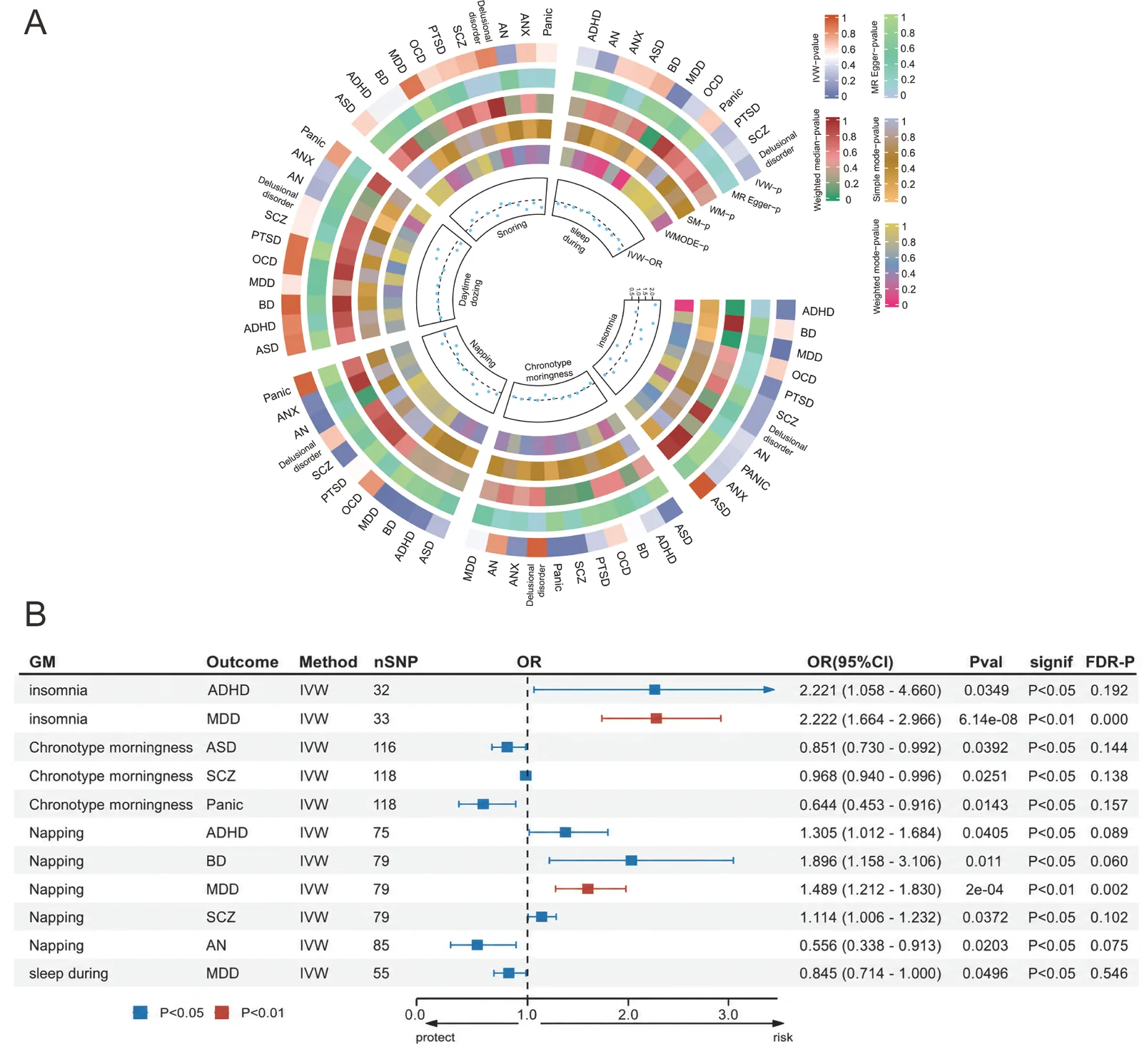
The results of forward Mendelian randomization analysis in IVW method. **(A)** It presents detailed results obtained using methods such as MR-Egger, weighted median, inverse variance weighted, simple mode, and weighted mode. **(B)** Causal associations between sleep disorders and psychiatric disorders in the forward MR analysis.

In the forward MR analyses, genetically predicted chronotype was associated with lower odds of ASD (OR = 0.85 [0.73–0.99], p = 0.039), panic disorder (OR 0.64 [0.45–0.91], p = 0.014), and SCZ (OR = 0.97 [0.940–0.996], p = 0.025); however, these associations did not remain statistically significant after FDR correction. Genetically predicted insomnia was associated with higher odds of ADHD and MDD; after FDR correction, only the association between insomnia and MDD remained statistically significant (FDR-adjusted p = 0.001). Genetically predicted napping was associated with higher odds of ADHD (OR = 1.30 [1.01–1.68], P = 0.040), BD (OR = 1.90 [1.16–3.10], p = 0.011), MDD (OR = 1.49 [1.21–1.83], p < 0.001), and SCZ (OR = 1.11 [1.01–1.23], p = 0.037); however, after FDR correction, only the association with MDD remained statistically significant (FDR-adjusted p = 0.001). Additionally, genetically predicted napping showed a nominal association with AN; however, this association did not remain significant after FDR correction. Genetically predicted sleep duration was associated with lower odds of MDD (OR = 0.84 [0.71–0.99], p = 0.049); however, this association did not remain significant after FDR correction. Detailed results are presented in **Figure 2** and **Supplementary Table 3**.

### 3.2 The results of sensitivity analyses

For associations showing statistical significance before multiple testing correction, sensitivity analyses were subsequently performed to evaluate the robustness of the findings. Cochran’s Q test and I² statistics did not indicate substantial heterogeneity among the genetic instruments. MR-Egger intercept analysis and MR-PRESSO global tests did not provide evidence of directional horizontal pleiotropy (p > 0.05). The MR-PRESSO outlier test did not identify significant outlier variants. Finally, leave-one-out analyses suggested that the observed associations were not driven by any single SNP (**Supplementary Figures 1-46**).

### 3.3 The results of reverse MR analysis

**Figure 3:**
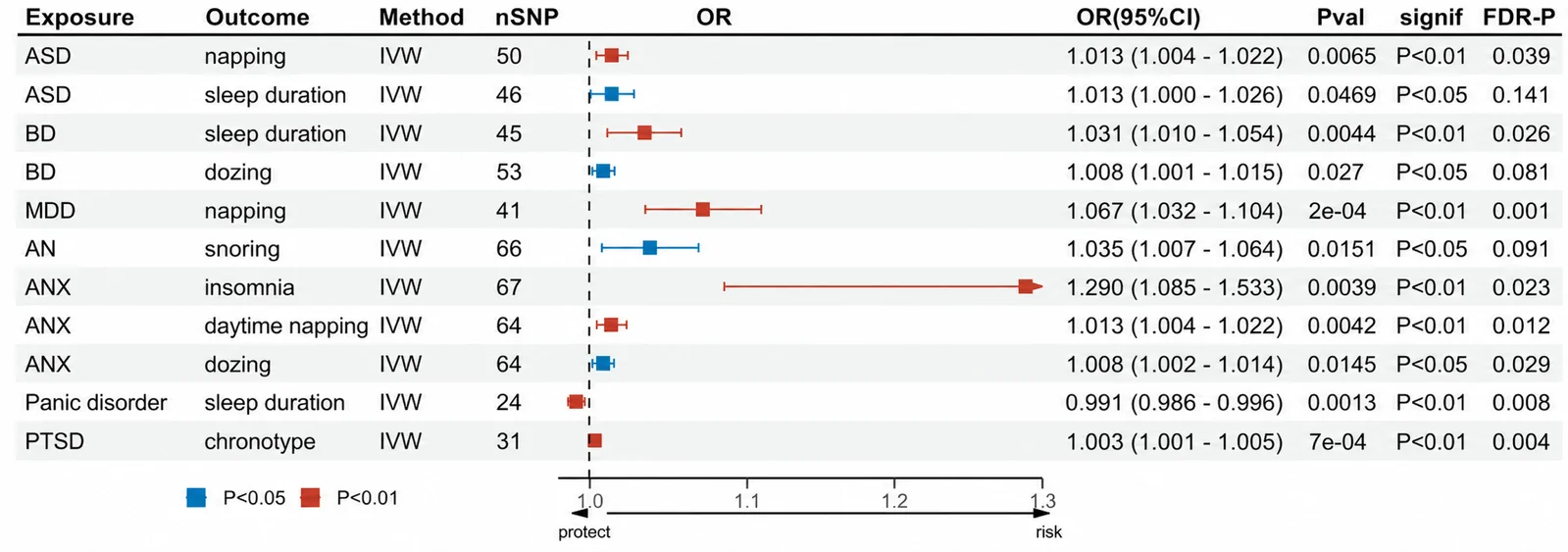
The results of forward Mendelian randomization analysis in IVW method. Causal associations between mental disorders and sleep disorders in reverse MR analyses.

In reverse MR analyses, sleep duration showed significant associations with MDD in both forward and reverse directions. To avoid overinterpretation of potentially bidirectional effects, this association was not included in the primary interpretation of the MR findings. Detailed results are presented in **Figure 3** and **Supplementary Table 4**.

### 3.4 Clinical observational analysis

**Figure 4:**
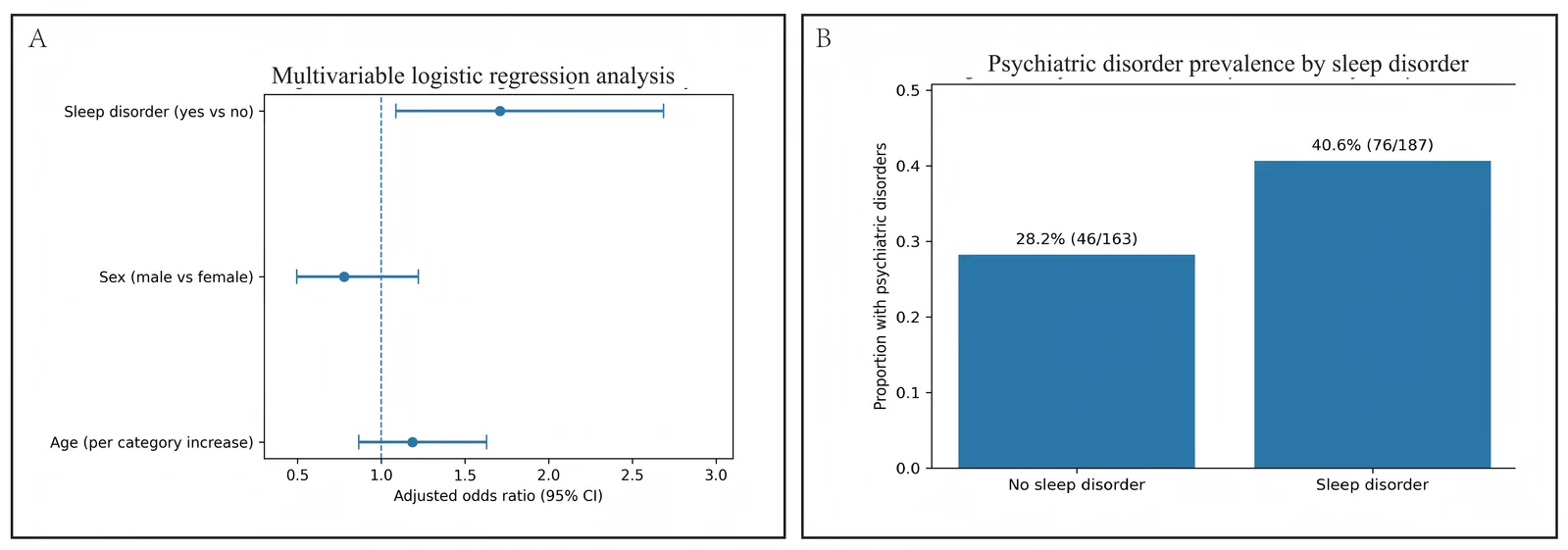
**(A)** Multivariable logistic regression of psychiatric disorders showing adjusted odds ratios (ORs) and 95% confidence intervals for sleep disorder, sex, and age category. **(B)** Prevalence of psychiatric disorders stratified by sleep disorder status (No sleep disorder: 46/163; Sleep disorder: 76/187).

Baseline characteristics of the clinical study population are summarized in **Table 1**. Among the 350 participants included in the analysis, 122 (34.9%) had psychiatric disorders. The prevalence of sleep disorders was significantly higher among participants with psychiatric disorders than among those without psychiatric disorders (62.3% vs 48.7%, p = 0.010). Crude analysis based on a 2×2 contingency table demonstrated that sleep disorder was positively associated with psychiatric disorders (OR = 1.74[1.11–2.73]) in **Table 2** and **Figure 4**. Results of the multivariable logistic regression analysis are presented in **Supplementary Table 5**. After adjustment for age and sex, sleep disorder remained independently associated with psychiatric disorders (OR = 1.71[1.09–2.69]). Sex and age were not significantly associated with psychiatric disorders in the adjusted model. The adjusted associations are illustrated in **Figure 4A**, and the prevalence of psychiatric disorders stratified by sleep disorders status is shown in **Figure 4B** indicating a higher prevalence among participants with sleep disorder.

**Table 1:** Baseline characteristics of the study population (n = 350).

Variable	Overall n (%)	No psychiatric disorder n (%)	Psychiatric disorder n (%)	P value
**Participants**	350 (100.0)	228 (65.1)	122 (34.9)	—
**Sleep disorder**				0.010
No	163 (46.6)	117 (51.3)	46 (37.7)	
Yes	187 (53.4)	111 (48.7)	76 (62.3)	
**Sex**				0.343
Female	181 (51.7)	122 (53.5)	59 (48.4)	
Male	169 (48.3)	106 (46.5)	63 (51.6)	
**Age group**				0.214†
<25 years	62 (17.7)	45 (19.7)	17 (13.9)	
25–60 years	198 (56.6)	121 (53.1)	77 (63.1)	
>60 years	90 (25.7)	62 (27.2)	28 (23.0)	

Values are presented as n (%). Percentages are column percentages. P values were calculated using χ² test unless otherwise indicated. † P value for trend.

**Table 2:** 2×2 contingency table (sleep disorders × mental disorders).

Sleep disorders	No psychiatric disorder	Psychiatric disorder	Total
No	117	46	163
Yes	111	76	187
Total	228	122	350

Crude odds ratio: 1.74 (95% CI 1.11–2.73).

## 4 Discussion

This study integrates MR analyses with clinical observational data to investigate potential associations between sleep-related traits and psychiatric disorders. A growing body of epidemiological evidence suggests that sleep disorders are associated with both overall psychiatric burden and specific psychiatric disorders (23). This study employed MR analyses to investigate potential associations between six sleep-related traits and eleven psychiatric disorders. Findings from forward MR analyses suggested that genetically predicted chronotype was associated with SCZ; however, no evidence of reverse association was observed in reverse MR analyses. Genetically predicted insomnia was associated with higher odds of MDD and ADHD; however, reverse MR analyses using MDD and ADHD as exposures did not identify significant reverse associations. These findings provide additional support for the directionality of the association between insomnia and depression, while residual uncertainty regarding causality remains. Notably, forward MR analyses suggested an association between genetically predicted daytime napping and MDD, and reverse MR analyses also identified an association in the opposite direction. Furthermore, genetically predicted longer sleep duration was associated with lower odds of MDD. Additionally, a retrospective observational study was conducted to evaluate the clinical association between sleep disorders and psychiatric disorders. The retrospective findings indicated that participants with sleep disorders exhibited a higher prevalence of psychiatric disorders (OR = 1.71[1.09–2.69], P = 0.020). The consistency between genetic evidence and observational clinical findings provides complementary support for the clinical relevance of these associations (20, 21). MR analyses may reduce the influence of residual confounding and reverse causality under specific assumptions, whereas clinical observational analyses provide information regarding real-world disease patterns. The consistency between these complementary approaches suggests that sleep disturbances may be involved in the development and clinical manifestation of psychiatric disorders. This highlights their potential clinical relevance as modifiable factors for mental health intervention (7, 22, 24, 25).

Findings from randomized clinical trials suggest that treatment of insomnia may improve outcomes related to incident and recurrent MDD among older adults with insomnia disorder. Recently, an MR analysis investigating insomnia and psychiatric disorders reported evidence supporting a potential causal association between insomnia and MDD (26). In reverse MR analyses, an inverse association was observed between MDD and daytime napping. Brain-derived neurotrophic factor (BDNF) is implicated in mediating plasticity changes associated with memory during sleep (27). Similarly, the findings of other randomized clinical trial indicate that treatment of insomnia with CBT-I has an overall benefit in the prevention of incident and recurrent major depression in older adults with insomnia disorder (28). In reverse MR, we obtained an inverse association between MDD and daytime napping. These findings suggest that daytime napping may represent a clinical characteristic of MDD rather than a direct causal consequence.

Our findings suggest that genetically predicted chronotype is associated with lower odds of ASD, panic disorder, and SCZ; however, no significant reverse associations were identified in reverse MR analyses. Regarding the relationship between chronotype and psychiatric disorders, genetic studies have suggested that circadian-related genes may contribute to understanding the biological links between sleep timing and mental health (29). Our findings are consistent with previous genetic evidence regarding the association between chronotype and SCZ, and additionally suggest potential associations between chronotype and ASD and panic disorder. However, the relationship between chronotype and SCZ remains incompletely understood, although an increasing number of studies have investigated their potential association (23, 30).

Genetically predicted insomnia was associated with higher odds of ADHD, and no significant reverse association was observed in reverse MR analyses, suggesting that the observed association may not be explained by reverse causality alone. In a cross-sectional study, comparisons among patients with insomnia (n = 60), patients without insomnia (n = 20), and healthy controls (n = 80) showed that scores for HAM-A, MADRS, DBAS, SRBQ (Sleep-Related Behavior Questionnaire), and PSAS were higher among patients with insomnia than among comparison groups. DBAS and SRBQ scores were positively correlated with insomnia severity. Dysfunctional beliefs regarding sleep (OR = 1.05[1.00-1.09]) and maladaptive behaviors related to sleep (OR = 1.02[1.00-1.05])predicted insomnia in patients with depression or anxiety (31, 32). Other longitudinal studies have also reported that insomnia was associated with subsequent ADHD onset (33). These findings are consistent with previous studies.

Genetically predicted longer sleep duration was associated with lower odds of MDD. Possible mechanisms may involve alterations in brain structure and function, changes in gene expression, abnormal protein accumulation or clearance, and dysregulation of related hormones and neurotransmitters. These biological changes may contribute to the observed associations between sleep disturbances and psychiatric disorders (34).

Psychiatric illnesses have also been reported to be associated with sleep disturbances. A cross-sectional study including psychiatric outpatients investigated the prevalence of sleep disorder symptoms among these individuals. The overall prevalence of sleep disorder symptoms in the psychiatric outpatient sample was 40.75% (163/400). The prevalence of symptoms related to narcolepsy, sleep breathing disorders, PLMS/RLS, circadian rhythm disorders, and parasomnias was 12.5%, 14.5%, 14.8%, 4.5%, and 13.8%, respectively (35). Potential underlying mechanisms, including activation of the hypothalamic-pituitary-adrenal (HPA) axis, dysfunction of the serotonin system, and overexpression of immune system peptides, may explain the phenomenon of inverse association (36). The sleep-related characteristics identified in this study suggest that, although some common features exist, sleep-related factors may involve distinct biological pathways across different psychiatric disorders. However, further research is needed to elucidate these mechanisms.

This study has several strengths. First, this study systematically investigated potential associations between multiple sleep-related traits and psychiatric disorders using a two-sample MR analysis method. Second, instrument strength was assessed using F-statistics, and all selected instrumental variables showed F-statistics greater than 10, suggesting sufficient instrument strength and reducing concerns regarding weak instrument bias. In addition, sensitivity analyses and heterogeneity assessments were performed to evaluate the robustness and consistency of the MR estimates. Third, a retrospective observational study was conducted in a Chinese clinical cohort, providing complementary clinical evidence regarding the clinical relevance of the genetic findings. Nevertheless, further multicenter and cross-population studies are needed to determine whether these findings can be generalized to other populations. However, several limitations should also be acknowledged. First, differences in GWAS sample characteristics, analytical strategies, and phenotype definitions across datasets may contribute to variability in the observed associations. Second, because detailed demographic information was unavailable in the GWAS datasets, subgroup analyses considering potential modifiers such as age and sex could not be performed. Third, the retrospective observational component was based on data from a single clinical center; therefore, future multicenter prospective studies are needed to further evaluate the reproducibility and external validity of these findings. Additionally, MR designs inherently have several limitations, including potential unmeasured confounding, horizontal pleiotropy, instrumental variable bias, and reverse causality. Although all retained genetic instruments had F statistics greater than 10, suggesting adequate instrument strength, weak-instrument bias cannot be completely excluded, particularly in analyses involving a relatively small number of SNPs. Although several methodological and sensitivity analyses were performed to evaluate the robustness of the estimates, these approaches cannot fully eliminate the possibility of residual bias. Finally, the clinical component of the present study was limited to a retrospective observational design. Future prospective, multicenter, and cross-population studies are needed to further clarify the temporal and potential causal relationships between sleep disorders and psychiatric disorders.

## 5 Conclusion

By integrating MR analyses with retrospective clinical observational data, this study provides genetic and clinical evidence supporting the association between sleep-related traits and psychiatric disorders. Genetically predicted insomnia and daytime napping showed potential causal associations with major depressive disorder. The retrospective clinical analysis further demonstrated an independent association between sleep disorders and psychiatric disorders in a real-world clinical setting. Collectively, these findings suggest that sleep disturbances may represent potentially modifiable factors associated with psychiatric disorders, highlighting the importance of sleep-related interventions for mental health prevention and management.

## Data Availability

The original contributions presented in the study are included in the article/**Supplementary Material**. Further inquiries can be directed to the corresponding author.
