# Correction: Genetic and clinical observational evidence for associations between sleep disorders and psychiatric disorders

**DOI:** 10.3389/fpsyt.2026.1988251

**Published:** 2026-09-14

**Authors:** Xin Wu, Mei Chang, Bingyi Song, Jiaxuan Li, Longyuan Li, Ziqian Yin, Zhouqing Chen, Zhong Wang

**Affiliations:** First Affiliated Hospital of Soochow University, Suzhou, China

**Keywords:** attention deficit hyperactivity disorder, insomnia, major depressive disorder, Mendelian randomization study, psychiatric disorders, retrospective study

Authors “Xin Wu, Mei Chang, and Bingyi Song” contributed equally to the authorship of this article. The initial publication failed to highlight this. A footnote has been added next to the names of these three authors to indicate that “These authors have contributed equally to this work”.

Author “Zhouqing Chen” was erroneously omitted as a corresponding author. The correct corresponding authors appear as follows:

“Zhouqing Chen, zqchen6@163.com; Zhong Wang, wangzhong761@163.com”

The original version of this article has been updated.

