# Supplementary material for: Genetic and clinical observational evidence for associations between sleep disorders and psychiatric disorders
Source: Front Psychiatry. 2026 Aug 14;17:1845263. doi: 10.3389/fpsyt.2026.1845263 (PMC13522940; doi:10.3389/fpsyt.2026.1845263)
Supplement: Supplementary file 1 [file DataSheet1.docx]

**Genetic and observational evidence for a causal association between sleep disorders and psychiatric disorders**

Xin Wu ^1, †^, Mei Chang ^1 †^, Bingyi Song^1†^, Jiaxuan Li ^1^, Longyuan Li ^1^, Ziqian Yin^1^, Zhouqing Chen ^1, *^, Zhong Wang ^1, *^

^1^ Department of Neurosurgery & Brain and Nerve Research Laboratory, The First Affiliated Hospital of Soochow University, Suzhou, Jiangsu Province, China

^†^ Xin Wu and Mei Chang contributed equally to this work.

*** Correspondence:**
Zhouqing Chen, Department of Neurosurgery, The First Affiliated Hospital of Soochow University, 188 Shizi Street, Suzhou 215006, China. Email address: zqchen6@163.com.

Zhong Wang, Department of Neurosurgery, The First Affiliated Hospital of Soochow University, 188 Shizi Street, Suzhou 215006, China. Email address: wangzhong761@163.com.

**Keywords:** Sleep disorder, insomnia, ADHD, major depression disorder, Mendelian randomization analysis

**Figure legends**

**Table S1: The detailed data information of outcomes.**

**Table S2: The detailed data information of exposures.**

**Table S3: The forward results of Mendelian randomization analyses.**

**Table S4: The reverse results of Mendelian randomization analyses.**

**Table S5: Multivariable logistic regression analysis of factors associated with psychiatric disorders**

**Figure S1: Scatter plot for the effect of chronotype on psychiatric disorder.**

**Figure S2: Scatter plot for the effect of insomnia on psychiatric disorder.**

**Figure S3: Scatter plot for the effect of sleep duration on MDD.**

**Figure S4: Scatter plot for the effect of napping on psychiatric disorder.**

**Figure S5: Leave-one-out plot for the effect of chronotype on ASD.**

**Figure S6: Leave-one-out plot for the effect of chronotype on SCZ.**

**Figure S7: Leave-one-out plot for the effect of chronotype on panic disorder.**

**Figure S8: Leave-one-out plot for the effect of insomnia on psychiatric disorder.**

**Figure S9: Leave-one-out plot for the effect of sleep duration on psychiatric disorder.**

**Figure S10: Leave-one-out plot for the effect of napping on ADHD.**

**Figure S11: Leave-one-out plot for the effect of napping on BD.**

**Figure S12: Leave-one-out plot for the effect of napping on MDD.**

**Figure S13: Leave-one-out plot for the effect of napping on SCZ.**

**Figure S14: Leave-one-out plot for the effect of napping on AN.**

**Figure S15: Funnel plot for the effect of chronotype on psychiatric disorder.**

**Figure S16: Funnel plot for the effect of insomnia on psychiatric disorder.**

**Figure S17: Funnel plot for the effect of sleep duration on psychiatric disorder.**

**Figure S18: Funnel plot for the effect of sleep duration on psychiatric disorder.**

**Figure S19: Forest plot for the effect of chronotype on ASD.**

**Figure S20: Forest plot for the effect of chronotype on panic disorder.**

**Figure S21: Forest plot for the effect of chronotype on SCZ.**

**Figure S22: Forest plot for the effect of insomnia on psychiatric disorder.**

**Figure S23: Forest plot for the effect of sleep duration on MDD.**

**Figure S24: Forest plot for the effect of napping on ADHD.**

**Figure S25: Forest plot for the effect of napping on BD.**

**Figure S26: Forest plot for the effect of napping on MDD.**

**Figure S27: Forest plot for the effect of napping on SCZ.**

**Figure S28: Forest plot for the effect of napping on AN.**

**Figure S29: Scatter plot for the effect of psychiatric disorder on sleep duration in reverse MR.**

**Figure S30: Leave-one-out plot for the effect of PTSD on chronotype in reverse MR.**

**Figure S31: Leave-one-out plot for the effect of ASD on napping in reverse MR.**

**Figure S32: Leave-one-out plot for the effect of ANX on napping in reverse MR.**

**Figure S33: Leave-one-out plot for the effect of ANX on insomnia in reverse MR.**

**Figure S34: Leave-one-out plot for the effect of MDD on napping in reverse MR.**

**Figure S35: Leave-one-out plot for the effect of ASD on sleep duration in reverse MR.**

**Figure S36: Leave-one-out plot for the effect of ASD on sleep duration in reverse MR.**

**Figure S37: Leave-one-out plot for the effect of panic disorder on sleep duration in reverse MR.**

**Figure S38: Funnel plot for the effect of psychiatric disorder on sleep duration in reverse MR.**

**Figure S39: Forest plot for the effect of PTSD on chronotype in reverse MR.**

**Figure S40：Forest plot for the effect of PTSD on chronotype in reverse MR.**

**Figure S41: Forest plot for the effect of ASD on napping in reverse MR.**

**Figure S42: Forest plot for the effect of MDD on napping in reverse MR.**

**Figure S43: Forest plot for the effect of ASD on sleep duration in reverse MR.**

**Figure S44: Forest plot for the effect of BD on sleep duration in reverse MR.**

**Figure S45: Forest plot for the effect of panic disorder on sleep duration in reverse MR.**

**Figure S46: Forest plot for the effect of ANX on napping in reverse MR.**

**Table S1: The detailed data information of outcomes.**

| **Outcomes** | **Consortium** | **Cases** | **Control** | **Population** | **Link** |
| --- | --- | --- | --- | --- | --- |
| ADHD | PGC | 38691 | 186843 | European | <https://pgc.unc.edu/for-researchers/download-results/> |
| MDD | PGC | 15771 | 178777 | European | <https://pgc.unc.edu/for-researchers/download-results/> |
| ASD | PGC | 18381 | 27969 | European | <https://pgc.unc.edu/for-researchers/download-results/> |
| SCZ | GWAS Catalog | 11602 | 36921 | European | <https://www.ebi.ac.uk/gwas/studies/GCST90275158> |
| OCD | PGC | 2688 | 7037 | European | <https://pgc.unc.edu/for-researchers/download-results/> |
| AN | PGC | 16992 | 55525 | European | <https://pgc.unc.edu/for-researchers/download-results/> |
| BD | PGC | 41917 | 371549 | European | <https://pgc.unc.edu/for-researchers/download-results/> |
| ANX | finnan | 21519 | 307558 | European | <https://storage.googleapis.com/finngen-public-data-r8/summary_stats/finngen_R8_F5_ALLANXIOUS.gz> |
| Panic disorder | PGC | 2248 | 7992 | European | <https://pgc.unc.edu/for-researchers/download-results/> |
| Delusional disorder | IEU | 1665 | 208674 | European | <https://gwas.mrcieu.ac.uk/datasets/finn-b-F5_DELUSIO/> |
| PTSD | PGC | 137136 | 1085746 | European | <https://pgc.unc.edu/for-researchers/download-results/> |

ADHD: Attention deficit hyperactivity disorder; ASD: Autism spectrum disorder; AN: Anorexia nervosa; ANX: Anxiety; SCZ: Schizophrenia; MDD: Major depression disorder;

OCD: Obsessive-compulsive disorder; DD: Delusional disorder; PD: Panic disorder; BD: Bipolar disorder; PTSD: Post-traumatic stress disorder.

**Table S2: The detailed data information of exposures.**

| **Exposure** | **Consortium** | **Population** | **Link** |
| --- | --- | --- | --- |
| Sleep duration | CMDKP | European | <https://sleep.hugeamp.org/downloads.html> |
| Snoring | CMDKP | European | <https://sleep.hugeamp.org/downloads.html> |
| Dozing | MRC-IEU | European | <https://gwas.mrcieu.ac.uk/datasets> |
| Napping | CMDKP | European | <https://sleep.hugeamp.org/downloads.html> |
| Chronotype | CMDKP | European | <https://sleep.hugeamp.org/downloads.html> |
| Insomnia | CMDKP | European | <https://sleep.hugeamp.org/downloads.html> |

CMDKP: Common metabolic diseases knowledge portal.

**Table S3: The forward results of Mendelian randomization analyses.**

| Exposure | Outcome | SNPs | Cochran's Q | | Pleiotropy | | | MR-PRESSO | | | | | | |
| --- | --- | --- | --- | --- | --- | --- | --- | --- | --- | --- | --- | --- | --- | --- |
|  |  |  | Q | P-value | MR- Egger intercept | SE | P-value | casual estimate^a^ | sd^a^ | P-value^a^ | Outliers | casual estimate^b^ | sd^b^ | P-value^b^ |
| Sleep duration | ADHD | 56 | 178.0 | 0.0000000 | 0.00123 | 0.00906 | 0.89230 | -0.1401 | 0.1618 | 0.3904 | 8 20 38 45 | -0.2269 | 0.1386 | 0.1078 |
|  | ASD | 59 | 105.6 | 0.0001343 | -0.01645 | 0.00863 | 0.06178 | -0.0777 | 0.1669 | 0.6433 | 13.0000 | -0.0016 | 0.1545 | 0.9917 |
|  | AN | 58 | 104.1 | 0.0001390 | -0.00155 | 0.00887 | 0.86154 | -0.2370 | 0.1673 | 0.1619 | 35.0000 | -0.1917 | 0.1603 | 0.2369 |
|  | ANX | 52 | 108.3 | 0.0000053 | 0.00669 | 0.00790 | 0.40115 | -0.0714 | 0.1483 | 0.6323 | 48.0000 | -0.0273 | 0.1426 | 0.8487 |
|  | SCZ | 57 | 57.0 | 0.4378665 | -0.00154 | 0.00143 | 0.28609 | 0.0234 | 0.0261 | 0.3728 | NA | NA | NA | NA |
|  | MDD | 55 | 254.5 | 0.0000000 | -0.00170 | 0.00444 | 0.70322 | -0.1686 | 0.0859 | 0.0548 | 2 7 12 39 45 | -0.1514 | 0.0687 | 0.0323 |
|  | OCD | 56 | 73.4 | 0.0495856 | 0.00382 | 0.01863 | 0.83847 | -0.3370 | 0.3583 | 0.3510 | NA | NA | NA | NA |
|  | Delusional disorder | 58 | 57.0 | 0.4766173 | 0.02400 | 0.01936 | 0.22031 | -0.4268 | 0.3653 | 0.2475 | NA | NA | NA | NA |
|  | Panic disorder | 56 | 47.7 | 0.7454086 | -0.00040 | 0.01734 | 0.98178 | -0.1553 | 0.3104 | 0.6188 | NA | NA | NA | NA |
|  | BD | 57 | 165.6 | 0.0000000 | -0.00143 | 0.00849 | 0.86673 | 0.0559 | 0.1529 | 0.7160 | 2 14 16 | -0.0115 | 0.1335 | 0.9314 |
|  | PTSD | 59 | 88.8 | 0.0057484 | 0.01076 | 0.00862 | 0.21714 | -0.1835 | 0.1638 | 0.2673 | 9.0000 | -0.1201 | 0.1547 | 0.4408 |
| Dozing  ukb-b-5776 | ADHD | 30 | 21.1 | 0.8541545 | -0.00788 | 0.00816 | 0.34213 | 0.0490 | 0.2114 | 0.8184 | NA | NA | NA | NA |
|  | ASD | 30 | 14.6 | 0.9881731 | 0.00073 | 0.01225 | 0.95259 | -0.0588 | 0.2598 | 0.8225 | NA | NA | NA | NA |
|  | AN | 30 | 62.6 | 0.0002883 | 0.01427 | 0.01791 | 0.43232 | 0.6417 | 0.5315 | 0.2370 | 5.0000 | 0.9348 | 0.4781 | 0.0606 |
|  | ANX | 30 | 50.1 | 0.0088188 | 0.00252 | 0.01281 | 0.84571 | -0.4197 | 0.3751 | 0.2724 | 22.0000 | -0.2511 | 0.3456 | 0.4735 |
|  | SCZ | 31 | 60.3 | 0.0008504 | -0.00402 | 0.00325 | 0.22490 | -0.0602 | 0.1003 | 0.5524 | 6 20 | -0.0708 | 0.0869 | 0.4226 |
|  | MDD | 28 | 72.9 | 0.0000043 | 0.00551 | 0.00641 | 0.39756 | 0.1096 | 0.1947 | 0.5780 | 21.0000 | 0.0443 | 0.1886 | 0.8163 |
|  | OCD | 28 | 4.5 | 0.9999997 | 0.02221 | 0.03239 | 0.49901 | 0.1152 | 0.3814 | 0.7649 | NA | NA | NA | NA |
|  | Delusional disorder | 30 | 22.0 | 0.8201642 | 0.02991 | 0.03099 | 0.34274 | 0.5446 | 0.8038 | 0.5034 | NA | NA | NA | NA |
|  | Panic disorder | 28 | 38.2 | 0.0745909 | -0.04713 | 0.04004 | 0.24977 | -0.3434 | 1.1867 | 0.7745 | NA | NA | NA | NA |
|  | BD | 30 | 125.2 | 0.0000000 | -0.00715 | 0.01777 | 0.69033 | 0.0369 | 0.5190 | 0.9439 | 2 8 11 16 29 30 | -0.0144 | 0.3814 | 0.9702 |
|  | PTSD | 30 | 7.1 | 0.9999900 | -0.00030 | 0.01338 | 0.98256 | 0.0502 | 0.1936 | 0.7973 | NA | NA | NA | NA |
| Snoring | ADHD | 21 | 7.1 | 0.9965050 | -0.00398 | 0.01522 | 0.79655 | 0.2359 | 0.1875 | 0.2229 | NA | NA | NA | NA |
|  | ASD | 24 | 8.4 | 0.9975569 | -0.00667 | 0.02182 | 0.76272 | -0.2205 | 0.2661 | 0.4157 | NA | NA | NA | NA |
|  | AN | 25 | 65.3 | 0.0000110 | 0.01123 | 0.03628 | 0.75970 | -1.0139 | 0.7135 | 0.1682 | 20.0000 | -0.5469 | 0.6139 | 0.3822 |
|  | ANX | 21 | 49.3 | 0.0002823 | -0.03891 | 0.02186 | 0.09098 | -0.2448 | 0.5542 | 0.6634 | 1.0000 | 0.0248 | 0.4880 | 0.9601 |
|  | SCZ | 22 | 20.7 | 0.4748529 | -0.01005 | 0.00390 | 0.01790 | 0.0343 | 0.0895 | 0.7053 | NA | NA | NA | NA |
|  | MDD | 21 | 40.6 | 0.0042091 | 0.00036 | 0.00883 | 0.96810 | 0.0289 | 0.2100 | 0.8919 | 17.0000 | 0.1214 | 0.1907 | 0.5321 |
|  | OCD | 20 | 2.6 | 0.9999971 | 0.01738 | 0.05214 | 0.74272 | -0.6226 | 0.4481 | 0.1808 | NA | NA | NA | NA |
|  | Delusional disorder | 29 | 39.6 | 0.0711317 | 0.09625 | 0.04665 | 0.04882 | -0.2374 | 1.1900 | 0.8433 | NA | NA | NA | NA |
|  | Panic disorder | 20 | 12.1 | 0.8792062 | 0.08894 | 0.05835 | 0.14481 | -0.7677 | 1.0263 | 0.4636 | NA | NA | NA | NA |
|  | BD | 22 | 64.8 | 0.0000024 | -0.01657 | 0.02429 | 0.50289 | 0.4140 | 0.5521 | 0.4617 | 8 9 11 | 0.7414 | 0.3733 | 0.0625 |
|  | PTSD | 25 | 9.0 | 0.9975787 | -0.01591 | 0.02063 | 0.44837 | 0.2055 | 0.2871 | 0.4811 | NA | NA | NA | NA |
| Insomnia | ADHD | 32 | 132.9 | 0.0000000 | -0.01540 | 0.01248 | 0.22691 | 0.7978 | 0.3782 | 0.0431 | 1 9 15 | 0.9973 | 0.2877 | 0.0017 |
|  | ASD | 33 | 90.5 | 0.0000002 | 0.00573 | 0.01518 | 0.70815 | -0.0121 | 0.4486 | 0.9786 | 7 15 16 | -0.1751 | 0.3830 | 0.6509 |
|  | AN | 33 | 79.8 | 0.0000059 | 0.02129 | 0.01439 | 0.14921 | 0.3794 | 0.4203 | 0.3733 | 26.0000 | 0.2256 | 0.3654 | 0.5414 |
|  | ANX | 35 | 61.1 | 0.0029661 | 0.00190 | 0.00803 | 0.81392 | 0.2591 | 0.2550 | 0.3168 | 34.0000 | 0.1503 | 0.2329 | 0.5230 |
|  | SCZ | 33 | 63.0 | 0.0008670 | 0.00167 | 0.00249 | 0.50810 | 0.0986 | 0.0735 | 0.1891 | NA | NA | NA | NA |
|  | MDD | 33 | 102.0 | 0.0000000 | 0.01199 | 0.00441 | 0.01063 | 0.7983 | 0.1474 | 0.0000 | 6 15 19 | 0.9264 | 0.1333 | 0.0000 |
|  | OCD | 33 | 48.0 | 0.0343669 | -0.03227 | 0.02526 | 0.21080 | 0.3856 | 0.7934 | 0.6303 | NA | NA | NA | NA |
|  | Delusional disorder | 37 | 47.7 | 0.0920159 | 0.01033 | 0.02652 | 0.69933 | -1.1070 | 0.8275 | 0.1894 | NA | NA | NA | NA |
|  | Panic disorder | 33 | 39.6 | 0.1678545 | -0.01082 | 0.02633 | 0.68384 | -0.7523 | 0.7794 | 0.3417 | NA | NA | NA | NA |
|  | BD | 33 | 85.8 | 0.0000008 | -0.00409 | 0.00995 | 0.68406 | -0.1648 | 0.2961 | 0.5817 | 7 22 | -0.1089 | 0.2402 | 0.6534 |
|  | PTSD | 33 | 30.1 | 0.5643097 | 0.01881 | 0.00946 | 0.05568 | 0.5279 | 0.2766 | 0.0653 | NA | NA | NA | NA |
| Napping | ADHD | 75 | 37.9 | 0.9998454 | 0.00102 | 0.00431 | 0.81411 | 0.2664 | 0.0930 | 0.0054 | NA | NA | NA | NA |
|  | ASD | 86 | 34.9 | 0.9999997 | 0.00672 | 0.00572 | 0.24354 | 0.2007 | 0.1135 | 0.0805 | NA | NA | NA | NA |
|  | AN | 85 | 172.6 | 0.0000000 | 0.00190 | 0.00829 | 0.81875 | -0.5874 | 0.2532 | 0.0227 | NA | NA | NA | NA |
|  | ANX | 87 | 151.6 | 0.0000164 | 0.00675 | 0.00610 | 0.27154 | 0.3427 | 0.1806 | 0.0611 | NA | NA | NA | NA |
|  | SCZ | 79 | 155.3 | 0.0000005 | 0.00010 | 0.00173 | 0.95368 | 0.1077 | 0.0517 | 0.0405 | 3.0000 | 0.0962 | 0.0502 | 0.0591 |
|  | MDD | 79 | 256.5 | 0.0000000 | 0.00429 | 0.00360 | 0.23792 | 0.3981 | 0.1052 | 0.0003 | 34 48 | 0.3047 | 0.0925 | 0.0015 |
|  | OCD | 80 | 19.0 | 1.0000000 | 0.01390 | 0.01595 | 0.38606 | -0.1203 | 0.2268 | 0.5972 | NA | NA | NA | NA |
|  | Delusional disorder | 88 | 116.0 | 0.0205165 | 0.00382 | 0.01727 | 0.82553 | -0.2275 | 0.5066 | 0.6545 | NA | NA | NA | NA |
|  | Panic disorder | 80 | 102.8 | 0.0371695 | -0.00136 | 0.01946 | 0.94448 | -0.0370 | 0.5610 | 0.9476 | NA | NA | NA | NA |
|  | BD | 79 | 305.2 | 0.0000000 | 0.00373 | 0.00851 | 0.66222 | 0.6400 | 0.2516 | 0.0130 | 19 21 23 32 46 | 0.4887 | 0.2113 | 0.0236 |
|  | PTSD | 86 | 20.8 | 1.0000000 | 0.00208 | 0.00619 | 0.73728 | 0.1241 | 0.0942 | 0.1914 | NA | NA | NA | NA |
| Chronotype | ADHD | 114 | 73.3 | 0.9985897 | 0.00248 | 0.00321 | 0.44193 | -0.0425 | 0.0375 | 0.2590 | NA | NA | NA | NA |
|  | ASD | 116 | 150.7 | 0.0143593 | -0.00451 | 0.00551 | 0.41422 | -0.1612 | 0.0782 | 0.0415 | NA | NA | NA | NA |
|  | AN | 114 | 207.2 | 0.0000002 | -0.00853 | 0.00653 | 0.19420 | 0.0249 | 0.0924 | 0.7878 | 21 62 | 0.0277 | 0.0871 | 0.7509 |
|  | ANX | 112 | 195.6 | 0.0000013 | 0.00330 | 0.00472 | 0.48601 | -0.1163 | 0.0685 | 0.0925 | 15 57 | -0.0742 | 0.0663 | 0.2657 |
|  | SCZ | 118 | 143.9 | 0.0460005 | 0.00047 | 0.00101 | 0.64696 | -0.0329 | 0.0147 | 0.0270 | NA | NA | NA | NA |
|  | MDD | 114 | 358.7 | 0.0000000 | 0.00132 | 0.00262 | 0.61632 | -0.0276 | 0.0379 | 0.4686 | 16 22 24 33 52 81 82 110 | -0.0322 | 0.0305 | 0.2940 |
|  | OCD | 116 | 26.9 | 1.0000000 | -0.00222 | 0.01217 | 0.85574 | 0.0866 | 0.0813 | 0.2891 | NA | NA | NA | NA |
|  | DD | 112 | 124.1 | 0.1865578 | 0.02203 | 0.01198 | 0.06858 | 0.0115 | 0.1763 | 0.9479 | NA | NA | NA | NA |
|  | PD | 118 | 103.0 | 0.8178837 | -0.00669 | 0.01295 | 0.60632 | -0.4398 | 0.1685 | 0.0103 | NA | NA | NA | NA |
|  | BD | 117 | 398.2 | 0.0000000 | 0.00455 | 0.00599 | 0.44872 | 0.0587 | 0.0851 | 0.4918 | 15 22 32 43 73 79 82 | 0.0880 | 0.0752 | 0.2449 |
|  | PTSD | 118 | 144.8 | 0.0413247 | 0.00340 | 0.00559 | 0.54410 | 0.0778 | 0.0803 | 0.3344 | NA | NA | NA | NA |

SNPs, Single-nucleotide polymorphisms; NA: Not available;

a: The results of MR-PRESSO are from raw data.

b: The results of MR-PRESSO are from Outlier-corrected.

ADHD: Attention deficit hyperactivity disorder; ASD: Autism spectrum disorder; AN: Anorexia nervosa; ANX: Anxiety; SCZ: Schizophrenia; MDD: Major depression disorder;

OCD: Obsessive-compulsive disorder; DD: Delusional disorder; PD: Panic disorder; BD: Bipolar disorder; PTSD: Post-traumatic stress disorder.

**Table S4: The reverse results of Mendelian randomization analyses**

| Exposure | Outcome | SNPs | Cochran's Q | | Pleiotropy | | | MR-PRESSO | | | | | | |
| --- | --- | --- | --- | --- | --- | --- | --- | --- | --- | --- | --- | --- | --- | --- |
|  |  |  | Q | P-value | MR- Egger intercept | SE | P-value | casual estimate^a^ | sd^a^ | P-value^a^ | Outliers | casual estimate^b^ | sd^b^ | P-value^b^ |
| ASD | Sleep duration | 46 | 76.8 | 0.0022 | 0.000076 | 0.0015 | 0.960 | 0.013 | 0.006 | 0.053 | 38 | 0.0099 | 0.0058 | 0.0939 |
|  | Snoring | 49 | 92.9 | 0.0001 | -0.004310 | 0.0031 | 0.175 | 0.006 | 0.014 | 0.676 | NA | NA | NA | NA |
|  | Dozing | 48 | 78.3 | 0.0028 | 0.001783 | 0.0006 | 0.005 | 0.005 | 0.003 | 0.085 | 23 39 | 0.0050 | 0.0024 | 0.0024 |
|  | Chronotype | 46 | 120.9 | 6.991431e-09 | 0.002098 | 0.0022 | 0.350 | -0.011 | 0.010 | 0.278 | 20 22 44 | -0.0162 | 0.0075 | 0.0359 |
|  | Napping | 50 | 166.4 | 1.078527e-14 | 0.001675 | 0.0011 | 0.120 | 0.013 | 0.005 | 0.009 | 25 31 38 | 0.0106 | 0.0037 | 0.0062 |
|  | Insomnia | 43 | 37.2 | 0.6809 | 0.007365 | 0.0127 | 0.566 | 0.039 | 0.050 | 0.441 | NA | NA | NA | NA |
| ADHD | Sleep duration | 12 | 30.7 | 0.0012 | 0.036122 | 0.0184 | 0.077 | 0.001 | 0.000 | 0.274 | 7 | 0.0002 | 0.0004 | 0.5781 |
|  | Snoring | 12 | 22.9 | 0.0183 | -0.051855 | 0.0345 | 0.163 | 0.001 | 0.001 | 0.341 | 10 | 0.0013 | 0.0006 | 0.0724 |
|  | Dozing | 12 | 22.9 | 0.0183 | -0.051855 | 0.0345 | 0.163 | 0.001 | 0.001 | 0.341 | 10 | 0.0013 | 0.0006 | 0.0724 |
|  | Chronotype | 12 | 48.5 | 1.183840e-06 | -0.015314 | 0.0319 | 0.641 | 0.001 | 0.001 | 0.171 | 7 10 | 0.0009 | 0.0006 | 0.1471 |
|  | Napping | 12 | 36.7 | 0.0001 | 0.016548 | 0.0115 | 0.182 | 0.000 | 0.000 | 0.358 | 3 9 | -0.0003 | 0.0002 | 0.2341 |
|  | Insomnia | 12 | 7.5 | 0.7609 | -0.020608 | 0.1344 | 0.881 | 0.001 | 0.002 | 0.677 | NA | NA | NA | NA |
| BD | Sleep duration | 45 | 159.2 | 5.991933e-15 | -0.006169 | -0.0062 | 0.084 | 0.031 | 0.011 | 0.007 | 1 16 17 24 | 0.0374 | 0.0088 | 0.0001 |
|  | Snoring | 43 | 95.1 | 5.378529e-06 | -0.000914 | 0.0059 | 0.878 | 0.011 | 0.018 | 0.538 | 35 | 0.0164 | 0.0173 | 0.3478 |
|  | Dozing | 53 | 109.5 | 5.636231e-06 | -0.000992 | 0.0012 | 0.404 | 0.008 | 0.003 | 0.031 | 1 | 0.0067 | 0.0034 | 0.0510 |
|  | Chronotype | 45 | 210.9 | 1.204834e-23 | 0.007402 | 0.0048 | 0.131 | -0.022 | 0.015 | 0.152 | 1 6 10 14 22 25 33 34 45 | -0.0199 | 0.0119 | 0.1020 |
|  | Napping | 52 | 242.7 | 9.309915e-27 | -0.000711 | 0.0021 | 0.736 | 0.736 | 0.006 | 0.075 | 2 4 13 16 18 20 34 48 | 0.0097 | 0.0050 | 0.0585 |
|  | Insomnia | 48 | 70.8 | 0.0140 | -0.023512 | 0.0248 | 0.348 | 0.124 | 0.073 | 0.093 | 26 | 0.1515 | 0.0672 | 0.0288 |
| SCZ | Sleep duration | 42 | 55.6 | 0.0632 | 0.000725 | 0.0014 | 0.610 | -0.013 | 0.031 | 0.678 | NA | NA | NA | NA |
|  | Snoring | 42 | 69.7 | 0.0034 | 0.003323 | 0.0031 | 0.294 | 0.072 | 0.070 | 0.310 | NA | NA | NA | NA |
|  | Dozing | 42 | 69.7 | 0.0034 | 0.003323 | 0.0031 | 0.294 | 0.072 | 0.070 | 0.310 | NA | NA | NA | NA |
|  | Chronotype | 42 | 55.5 | 0.0652 | 0.002335 | 0.0016 | 0.162 | -0.022 | 0.036 | 0.547 | NA | NA | NA | NA |
|  | Napping | 44 | 108.3 | 1.516133e-07 | 0.000570 | 0.0010 | 0.564 | 0.010 | 0.022 | 0.642 | 8 26 | 0.0146 | 0.0168 | 0.3893 |
|  | Insomnia | 45 | 46.6 | 0.3661 | 0.005522 | 0.0138 | 0.690 | -0.520 | 0.290 | 0.080 | NA | NA | NA | NA |
| MDD | Sleep duration | 39 | 260.1 | 6.718627e-35 | -0.008086 | 0.0054 | 0.144 | 0.022 | 0.035 | 0.535 | 2 9 15 19 20 23 26 27 28 38 | 0.0084 | 0.0226 | 0.7133 |
|  | Snoring | 41 | 121.8 | 3.420605e-10 | 0.005667 | 0.0074 | 0.450 | -0.032 | 0.047 | 0.497 | 3 41 | 0.0059 | 0.0402 | 0.8841 |
|  | Dozing | 41 | 121.7868 | 3.420605e-10 | 0.005667 | 0.0074 | 0.450 | -0.032 | 0.047 | 0.497 | 3 41 | 0.0059 | 0.0402 | 0.8841 |
|  | Chronotype | 39 | 203.8 | 1.449535e-24 | -0.009728 | 0.0056 | 0.093 | 0.010 | 0.037 | 0.786 | 5 6 8 11 18 19 23 36 39 | 0.0124 | 0.0235 | 0.6026 |
|  | Napping | 41 | 241.9 | 1.053643e-30 | -0.005686 | 0.0026 | 0.036 | 0.065 | 0.017 | 0.001 | 19 20 24 40 | 0.0616 | 0.0120 | 0.0000 |
|  | Insomnia | 40 | 52.4 | 0.0737 | 0.015391 | 0.0315 | 0.628 | 0.147 | 0.168 | 0.389 | NA | NA | NA | NA |
| DD | Sleep duration | 22 | 41.2 | 0.0052 | 0.000580 | 0.0015 | 0.699 | 0.000 | 0.003 | 0.976 | 12 22 | -0.0002 | 0.0023 | 0.9388 |
|  | Snoring | 24 | 38.2 | 0.0242 | -0.003623 | 0.0027 | 0.185 | 0.005 | 0.006 | 0.432 | NA | NA | NA | NA |
|  | Dozing | 24 | 34.8 | 0.0541 | -0.000778 | 0.0006 | 0.178 | -0.001 | 0.001 | 0.476 | NA | NA | NA | NA |
|  | Chronotype | 24 | 75.8 | 1.504242e-07 | 0.000448 | 0.0022 | 0.843 | 0.002 | 0.005 | 0.652 | 14 16 20 | 0.0056 | 0.0039 | 0.1638 |
|  | Napping | 24 | 35.7 | 0.0442 | -0.000505 | 0.0007 | 0.470 | -0.001 | 0.002 | 0.587 | NA | NA | NA | NA |
|  | Insomnia | 24 | 21.1 | 0.5764 | -0.006731 | 0.0148 | 0.654 | 0.016 | 0.033 | 0.628 | NA | NA | NA | NA |
| AN | Sleep duration | 63 | 90.8 | 0.0100 | 0.000580 | 0.0017 | 0.731 | -0.006 | 0.005 | 0.265 | 11 58 | -0.0096 | 0.0046 | 0.0402 |
|  | Snoring | 66 | 179.6 | 1.118053e-12 | -0.001997 | 0.0047 | 0.671 | 0.034 | 0.014 | 0.018 | 6 52 | 0.0236 | 0.0124 | 0.0620 |
|  | Dozing | 66 | 166.6 | 7.128204e-11 | -0.000182 | 0.0010 | 0.856 | 0.001 | 0.003 | 0.706 | 1 12 | 0.0009 | 0.0028 | 0.7419 |
|  | Chronotype | 63 | 158.4 | 2.221507e-10 | 0.005498 | 0.0025 | 0.035 | 0.009 | 0.008 | 0.264 | 33 60 61 | 0.0072 | 0.0075 | 0.3441 |
|  | Napping | 66 | 217.1 | 2.912547e-18 | -0.000521 | 0.0013 | 0.700 | 0.000 | 0.004 | 0.960 | 2 12 58 | 0.0007 | 0.0030 | 0.8098 |
|  | Insomnia | 64 | 86.3 | 0.0275 | -0.015869 | 0.0169 | 0.352 | 0.044 | 0.052 | 0.396 | NA | NA | NA | NA |
| ANX | Sleep duration | 52 | 157.7 | 7.978495e-13 | -0.000537 | 0.0021 | 0.800 | 0.009 | 0.012 | 0.461 | 10 15 40 | 0.0113 | 0.0100 | 0.2639 |
|  | Snoring | 62 | 86.4 | 0.0180 | -0.001926 | 0.0027 | 0.475 | -0.017 | 0.015 | 0.248 | 59 | -0.0219 | 0.0139 | 0.1203 |
|  | Dozing | 64 | 90.7 | 0.0127 | 0.000471 | 0.0006 | 0.422 | 0.008 | 0.003 | 0.017 | NA | NA | NA | NA |
|  | Chronotype | 62 | 151.6 | 1.137626e-09 | -0.000456 | 0.0020 | 0.823 | -0.016 | 0.011 | 0.154 | 52 61 | -0.0085 | 0.0100 | 0.4014 |
|  | Napping | 64 | 127.0 | 3.267583e-06 | 0.000471 | 0.0008 | 0.565 | 0.013 | 0.004 | 0.006 | NA | NA | NA | NA |
|  | Insomnia | 67 | 59.5 | 0.7020 | 0.004721 | 0.0168 | 0.780 | 0.254 | 0.084 | 0.003 | NA | NA | NA | NA |
| PD | Sleep duration | 24 | 25.8 | 0.3082 | 0.001662 | 0.0018 | 0.364 | -0.009 | 0.003 | 0.004 | NA | NA | NA | NA |
|  | Snoring | 26 | 42.4 | 0.0163 | -0.004099 | 0.0044 | 0.362 | -0.004 | 0.007 | 0.567 | 12 | -0.0084 | 0.0059 | 0.1706 |
|  | Dozing | 26 | 26.2 | 0.3989 | 0.000068 | 0.0008 | 0.932 | -0.001 | 0.001 | 0.302 | NA | NA | NA | NA |
|  | Chronotype | 25 | 71.2 | 1.446233e-06 | -0.001168 | 0.0035 | 0.742 | -0.003 | 0.005 | 0.610 | 11 | 0.0006 | 0.0045 | 0.8916 |
|  | Napping | 26 | 37.1 | 0.0564 | 0.000158 | 0.0011 | 0.885 | -0.002 | 0.002 | 0.344 | NA | NA | NA | NA |
|  | Insomnia | 24 | 15.9 | 15.8630 | -0.0151325 | 0.0183 | 0.416 | -0.013 | 0.022 | 0.575 | NA | NA | NA | NA |
| OCD | Sleep duration | 19 | 20.5 | 0.3074 | 0.000727 | 0.0033 | 0.827 | -0.001 | 0.001 | 0.479 | NA | NA | NA | NA |
|  | Snoring | 18 | 22.2 | 0.1780 | -0.001796 | 0.0074 | 0.811 | 0.002 | 0.002 | 0.168 | NA | NA | NA | NA |
|  | Dozing | 20 | 31.3 | 0.0370 | 0.000769 | 0.0017 | 0.655 | 0.000 | 0.000 | 0.288 | NA | NA | NA | NA |
|  | Chronotype | 19 | 19.2 | 0.3788 | -0.007883 | 0.0035 | 0.040 | 0.000 | 0.001 | 0.886 | NA | NA | NA | NA |
|  | Napping | 20 | 24.8 | 0.1679 | 0.001543 | 0.0017 | 0.388 | 0.000 | 0.000 | 0.367 | NA | NA | NA | NA |
|  | Insomnia | 18 | 17.6 | 0.4154 | 0.046928 | 0.0335 | 0.180 | -0.007 | 0.008 | 0.399 | NA | NA | NA | NA |
| PTSD | Sleep duration | 31 | 64.8 | 0.0002 | -0.005325 | 0.0083 | 0.527 | -0.001 | 0.001 | 0.191 | 16 28 | 0.0000 | 0.0008 | 0.9637 |
|  | Snoring | 29 | 34.7 | 0.1775 | 0.018999 | 0.0131 | 0.158 | 0.000 | 0.002 | 0.804 | NA | NA | NA | NA |
|  | Dozing | 31 | 42.3 | 0.0669 | 0.001765 | 0.0031 | 0.569 | 0.000 | 0.000 | 0.717 | NA | NA | NA | NA |
|  | Chronotype | 31 | 36.5 | 0.1929 | 0.015301 | 0.0069 | 0.034 | 0.003 | 0.001 | 0.002 | NA | NA | NA | NA |
|  | Napping | 31 | 32.0 | 0.3664 | -0.004384 | 0.0030 | 0.161 | 0.000 | 0.000 | 0.548 | NA | NA | NA | NA |
|  | Insomnia | 30 | 20.9 | 0.8623 | -0.021717 | 0.0523 | 0.681 | 0.005 | 0.006 | 0.408 | NA | NA | NA | NA |

SNPs, Single-nucleotide polymorphisms; NA: Not available;

a: The results of MR-PRESSO are from raw data.

b: The results of MR-PRESSO are from Outlier-corrected.

ADHD: Attention deficit hyperactivity disorder; ASD: Autism spectrum disorder; AN: Anorexia nervosa; ANX: Anxiety; SCZ: Schizophrenia; MDD: Major depression disorder;

OCD: Obsessive-compulsive disorder; DD: Delusional disorder; PD: Panic disorder; BD: Bipolar disorder; PTSD: Post-traumatic stress disorder.

**Table S5: Multivariable logistic regression analysis of factors associated with psychiatric disorders**

| Variable | Adjusted OR | 95% CI | P value |
| --- | --- | --- | --- |
| Sleep disorder (yes vs no) | 1.71 | 1.09–2.69 | 0.020 |
| Sex (male vs female) | 0.78 | 0.49–1.22 | 0.275 |
| Age (per category increase) | 1.19 | 0.86–1.63 | 0.289 |

OR=odds ratio；CI=confidence interval.

The model was adjusted for age and sex.

**Figure S1: Scatter plot for the effect of chronotype on psychiatric disorder.**

**Figure S2: Scatter plot for the effect of insomnia on psychiatric disorder.**

**Figure S3: Scatter plot for the effect of sleep duration on MDD.**

**Figure S4: Scatter plot for the effect of napping on psychiatric disorder.**

**Figure S5: Leave-one-out plot for the effect of chronotype on ASD.**

**Figure S6: Leave-one-out plot for the effect of chronotype on SCZ.**

**Figure S7: Leave-one-out plot for the effect of chronotype on panic disorder.**

**Figure S8: Leave-one-out plot for the effect of insomnia on psychiatric disorder.**

**Figure S9: Leave-one-out plot for the effect of sleep duration on psychiatric disorder.**

**Figure S10: Leave-one-out plot for the effect of napping on ADHD.**

**Figure S11: Leave-one-out plot for the effect of napping on BD.**

**Figure S12: Leave-one-out plot for the effect of napping on MDD.**

**Figure S13: Leave-one-out plot for the effect of napping on SCZ.**

**Figure S14: Leave-one-out plot for the effect of napping on AN.**

**Figure S15: Funnel plot for the effect of chronotype on psychiatric disorder.**

**Figure S16: Funnel plot for the effect of insomnia on psychiatric disorder.**

**Figure S17: Funnel plot for the effect of sleep duration on MDD.**

**Figure S18: Funnel plot for the effect of napping on psychiatric disorder.**

**Figure S19: Forest plot for the effect of chronotype on ASD.**

**Figure S20: Forest plot for the effect of chronotype on panic disorder.**

**Figure S21: Forest plot for the effect of chronotype on SCZ.**

**Figure S22: Forest plot for the effect of insomnia on psychiatric disorder.**

**Figure S23: Forest plot for the effect of sleep duration on MDD.**

**Figure S24: Forest plot for the effect of napping on ADHD.**

**Figure S25: Forest plot for the effect of napping on BD.**

**Figure S26: Forest plot for the effect of napping on MDD.**

**Figure S27: Forest plot for the effect of napping on SCZ.**

**Figure S28: Forest plot for the effect of napping on AN.**

**Figure S29: Scatter plot for the effect of psychiatric disorder on sleep disorder in reverse MR.**

**Figure S30: Leave-one-out plot for the effect of PTSD on chronotype in reverse MR.**

**Figure S31: Leave-one-out plot for the effect of ASD on napping in reverse MR.**

**Figure S32: Leave-one-out plot for the effect of ANX on napping in reverse MR.**

**Figure S33: Leave-one-out plot for the effect of ANX on insomnia in reverse MR.**

**Figure S34: Leave-one-out plot for the effect of MDD on napping in reverse MR.**

**Figure S35: Leave-one-out plot for the effect of ASD on sleep duration in reverse MR.**

**Figure S36: Leave-one-out plot for the effect of ASD on sleep duration in reverse MR.**

**Figure S37: Leave-one-out plot for the effect of panic disoeder on sleep duration in reverse MR.**

**Figure S38: Funnel plot for the effect of psychiatric disorder on sleep duration in reverse MR.**

**Figure S39: Forest plot for the effect of PTSD on chronotype in reverse MR.**

**Figure S40：Forest plot for the effect of PTSD on chronotype in reverse MR.**

**Figure S41: Forest plot for the effect of ASD on napping in reverse MR.**

**Figure S42: Forest plot for the effect of MDD on napping in reverse MR.**

**Figure S43: Forest plot for the effect of ASD on sleep duration in reverse MR.**

**Figure S44: Forest plot for the effect of BD on sleep duration in reverse MR.**

**Figure S45: Forest plot for the effect of panic disorder on sleep duration in reverse MR.**

**Figure S46: Forest plot for the effect of ANX on napping in reverse MR.**
